# Cardiac Damage Staging, Moderate Aortic Stenosis, and the Impact of Aortic Valve Replacement

**DOI:** 10.1016/j.jacadv.2025.102348

**Published:** 2025-11-15

**Authors:** Evan K. Harmon, Travis Howard, Larisa G. Tereshchenko, Amar Krishnaswamy, Grant W. Reed, Elizabeth Ghandakly, James J.Y. Yun, Marijan Koprivanac, Ankur Kalra, Serge C. Harb, Samir R. Kapadia, Rishi Puri

**Affiliations:** a Department of Cardiovascular Medicine, Cleveland Clinic, Cleveland, Ohio, USA; b Department of Quantitative Health Sciences, Lerner Research Institute, Cleveland, Ohio, USA; c Department of Cardiac Surgery, Cleveland Clinic, Cleveland, Ohio, USA; d Department of Medicine, SUNY Upstate Medical University, Syracuse, New York, USA

**Keywords:** aortic valve replacement, cardiac damage, moderate aortic stenosis

## Abstract

**Background:**

Cardiac damage stage predicts clinical outcomes in severe aortic stenosis (AS), yet its validation in moderate AS and natural history preaortic valve replacement (AVR) and post-AVR is poorly understood.

**Objectives:**

This paper sought to: 1) determine whether index cardiac damage stage predicts cardiovascular outcomes in patients with moderate AS; and 2) describe the impact of AVR on cardiac damage stage over time.

**Methods:**

This retrospective single-center cohort study stratified patients with moderate AS according to index cardiac damage stage. We assessed whether cardiac damage stage predicted a primary composite outcome of all-cause death and heart failure (HF) hospitalization and a secondary outcome of AVR. Changes in stage over time were also assessed.

**Results:**

Among 688 patients (median follow-up of 3.6 years), baseline cardiac damage stages 0, 1, 2, and 3 to 4 were evident in 25.1%, 24.1%, 41.6%, and 9.2%, respectively. Escalating baseline stage conferred an increased risk of the composite primary endpoint (incidence per 1,000 person years, stage 0: 52 [37, 72]; stage 1: 100 [77, 129], stage 2: 94 [77, 116]; stages 3 to 4: 255 [185, 352]; HR (95% CI); *P* < 0.001], which was mirrored in survival analyses (all-cause death and HF hospitalization: *P*_log-rank_ < 0.001). The net result of awaiting AVR (median time 3.1 years) was an accumulation of cardiac damage, which partially resolved at 1-year.

**Conclusions:**

Cardiac damage stage is associated with an increasing risk of all-cause mortality and HF hospitalization in patients with moderate AS. The degree of damage accumulates as patients await AVR, which partially recovers post-AVR.

Patients with severe aortic stenosis (AS) harbor a Class I indication for aortic valve (AV) replacement (AVR),^1^ and available data suggest that AVR is beneficial in severe AS patients irrespective of symptom presence.^2^^,^^3^ However, an increasing body of evidence demonstrates moderate AS to portend an almost equivalent adverse event rate compared with untreated severe AS, particularly in the presence of impaired diastolic function and/or elevated B-natriuretic peptide levels.^4^, ^5^, ^6^, ^7^ Current guidelines recommend these patients undergo surveillance imaging every 1 to 2 years, and only recommend AVR for moderate AS if patients are to undergo open heart surgery for a separate indication (Class 2B).^1^ These relatively limited recommendations reflect the paucity of knowledge in the optimal management strategy of these patients.

AS rarely exists in isolation;^8^ nevertheless, its presence and interactions with a range of concomitant comorbidities substantially impacts cardiac structure, function, and clinical outcomes. Formally staging the extent of underlying cardiac damage in the presence of AS at the time of its diagnosis has consistently predicted all-cause mortality in both symptomatic^9^, ^10^, ^11^ and asymptomatic^12^ patients with moderate-to-severe AS. However, less is known about the impact of the extent of baseline cardiac damage stage in true moderate AS patients, the time course effects, and the impact of AVR on cardiac damage staging. A better understanding of the vulnerable moderate AS phenotype may allow us to implement earlier, more aggressive therapeutic measures, including earlier AVR, in such individuals.

Accordingly, we sought to determine the prognostic impact of cardiac damage stage at the time of moderate AS diagnosis on all-cause death and heart failure (HF) hospitalization. We secondarily aimed to describe how cardiac damage stage changes over time in moderate AS patients who undergo AVR, as well as the impact of AVR per se on cardiac damage stage.

## Methods

### Study population, definitions, and end points

This was a single-center retrospective cohort study performed within the Cleveland Clinic Health System. Approval for the current study was obtained from the Cleveland Clinic Institutional Review Board. The echocardiographic database was reviewed for echocardiograms performed from January 2016 through November 2020 in both the inpatient and outpatient settings. Only patients ≥18 years of age and with a complete 2D and Doppler echocardiographic assessment were included. Echocardiographic exclusion criteria included moderate or greater aortic insufficiency, moderate or greater non-AV stenosis, and prior AV surgery. We identified patients with moderate AS, defined as AV area 1.0 to 1.5 cm^2^, mean AV gradient 20 to 39 mm Hg, and peak aortic transvalvular velocity <4 m/s. For patients with multiple qualifying echocardiograms, the initial echocardiogram meeting moderate AS criteria was designated at the study start date (hereafter referred to as “baseline echocardiogram”), as has been validated previously.^5^ For patients in atrial fibrillation (AF) at the time of their echocardiogram, multiple beat averaging was used to determine peak aortic transvalvular velocity as supported by current guideline recommendations.^13^

Demographic, laboratory, follow-up, and surgical data were extracted from the electronic medical record using an Epic data warehouse, Current Procedural Terminology codes, and *International Classification of Diseases* (ICD) codes. For patients who ultimately underwent AVR, data from 1 pre-AVR echocardiogram and 2 post-AVR echocardiograms were obtained. To be included, the pre-AVR echocardiogram needed to occur within 1 to 3 months before AVR (hereafter referred to as “pre-AVR echocardiogram”). Data from 2 separate post-AVR echocardiograms occurring between 1 to 3 months postoperatively as well as 1 to 3 years postoperatively were also collected (hereafter referred to as “1-month echocardiogram” and “1-year echocardiogram”). Procedural AVR data were obtained using ICD-10 codes as well as independent review of surgical reports.

Cardiac damage staging refers to the extent of extra-aortic valvular structural and hemodynamic changes occurring as a consequence of a patient’s AV disease and has been described in detail elsewhere.^9^ Briefly, patients were assigned to 1 of 5 independent cardiac damage stages (stages 0-4) based on echocardiographic and clinical data available at the time of baseline echocardiogram demonstrating moderate AS. Stage 1 is defined as left ventricular (LV) damage, requiring the presence of LV hypertrophy (LV mass index >95 g/m^2^ for women, >115 g/m^2^ for men), severe LV diastolic dysfunction (E/e’ >14), or LV systolic dysfunction (left ventricular ejection fraction [LVEF] <50%).^9^^,^^14^ Stage 2 is defined as left atrial (LA) and/or mitral valve (MV) damage, requiring the presence of LA dilatation (LA volume indexed to body surface area >34 mL/m^2^), moderate (2+) or greater mitral regurgitation (MR), or the presence of AF. Stage 3 is defined as pulmonary vasculature and/or tricuspid valve damage, requiring systolic pulmonary hypertension (estimated pulmonary artery systolic pressure ≥60 mm Hg) or moderate (2+) or greater tricuspid regurgitation (TR). Stage 4 is defined as right ventricular (RV) damage, requiring moderate or greater decrease in RV systolic function. Individual patients were assigned to the highest cardiac damage stage for which they met at least one of the prespecified criteria. Patients meeting none of the criteria were not considered to have any extra-AV cardiac damage and were assigned to stage 0. Given the relatively low number of patients anticipated to have stage 4 cardiac damage resulting from moderate AS, patients in stages 3 to 4 were combined for subsequent analyses. For patients who underwent AVR, cardiac damage stages were assigned at baseline, at pre-AVR echocardiogram, at 1-month post-AVR echocardiogram, and at 1-year post-AVR echocardiogram.

The primary endpoint was a composite of all-cause death or HF hospitalization. HF hospitalization was included if an ICD code or discharge diagnosis adjudicated acute decompensated HF. We sought to understand whether cardiac damage stage at time of moderate AS diagnosis was associated with the primary endpoint. Our secondary endpoint included time to AVR, and the AVR endpoint itself included both transcatheter AVR (TAVR) as well as surgical AVR (SAVR). Among patients who underwent AVR, we aimed to understand how cardiac damage stage changed over time, both leading up to and following AVR.

### Statistical analyses

In descriptive baseline statistics, continuous variables that were normally distributed are presented as mean ± SD and were compared using t-test, whereas categorical variables were compared using chi-square test. Variables that were not normally distributed are presented as median (IQR) and were compared using Wilcoxon rank-sum test. Multiple normally-distributed groups were compared using analysis of variance.

For all analyses we combined cardiac damage stages 3 and 4 given the relatively small number of patients in each of these groups (n = 55 for stage 3, n = 8 for stage 4). We performed survival analyses using both univariable and multivariable Cox-proportional hazard regression to calculate the unadjusted HR with 95% CI. Incidence rates for primary and secondary outcomes are reported in person-time (per 1,000 patient-years of follow-up). Multivariable Cox regression model was adjusted for age, sex, presence of diabetes, coronary artery disease, prior myocardial infarction, chronic obstructive pulmonary disease (COPD), end-stage renal disease, AF, prior stroke, moderate or greater MR, and moderate or greater TR. In addition, to account for AVR as a competing time-dependent event, we constructed a cause-specific Cox regression model for the primary outcome, censoring at the time of AVR.

Transition probabilities were calculated to assess the change in cardiac damage over time in patients who underwent AVR. These analyses were descriptive in nature given our lack of a comparator group. For patients who underwent AVR, we estimated 2 Markov transition matrices: 1) for the probability of change in cardiac damage stage from the time of diagnosis to the time of AVR; and 2) for the probability of change after AVR (at 1-month, and at 1-year, as compared with pre-AVR). Transitions from nonmissing to missing or from missing to nonmissing values were not counted; the analysis did not normalize for missing periods. The Markov transition matrix was fully rectangularized. Panel-data line plots illustrated the change of stage over time. All statistical analyses were performed using STATA (version 18.0, StataCorp).

## Results

### Baseline characteristics and cardiac damage staging

A total of 688 patients were included in the study. Of these patients, 173 (25.1%) had no cardiac damage (stage 0), 166 (24.1%) had LV damage (stage 1), 286 (41.6%) had LA or MV damage (stage 2), 55 (8.0%) had pulmonary vasculature or tricuspid valve damage (stage 3), and 8 (1.2%) had RV damage (stage 4). The prevalence of cardiac damage stages and their individual components in the cohort are described in Table 1. Overall, the cohort was predominantly male (57.1%) with a mean age of 72.5 years and a median BMI of 30.0 kg/m^2^. The commonest medical comorbidities were hypertension (85.6%), hyperlipidemia (75.1%), coronary artery disease (51.6%), AF (41.4%), and diabetes (37.1%). Escalating cardiac damage stage was associated with increasing age and a higher prevalence of hyperlipidemia, AF, prior stroke, COPD, and advanced kidney disease. A full description of the cohort demographics is provided in Table 2.

**Table 1:** Prevalence of Cardiac Damage Stages and Their Individual Components

Stage 0: no cardiac damage	173/688 (25.1)
Stage 1: LV damage	166/688 (24.1)
Stage 2: left atrial or mitral valve damage	286/688 (41.6)
Stage 3: pulmonary vasculature or tricuspid valve damage	55/688 (8.0)
Stage 4: RV damage	8/688 (1.2)
Individual components of cardiac damage	
Stage 1: LV damage	166/688 (24.1)
Increased LV mass index	203/688 (29.5)
M (>115 g/m^2^)	101/393 (25.7)
F (>95 g/m^2^)	102/295 (34.6)
E/e’ >14	282/688 (41.0)
LV ejection fraction <50%	51/688 (7.4)
Stage 2: left atrial or mitral valve damage	286/688 (41.6)
Indexed left atrial volume >34 mL/m^2^	237/688 (34.4)
Moderate-severe mitral regurgitation	50/688 (7.3)
Atrial fibrillation	146/688 (21.2)
Stage 3: pulmonary vasculature or tricuspid valve damage	55/688 (8.0)
Systolic pulmonary hypertension ≥60 mm Hg	26/688 (3.8)
Moderate-severe tricuspid regurgitation	49/688 (7.1)
Stage 4: RV damage	8/688 (1.2)
Moderate-severe right ventricular dysfunction	8/688 (1.2)

Values are n/N (%).

LV = left ventricular; RV = right ventricular.

**Table 2:** Clinical Characteristics of the Total Patient Population

	Total Population (N = 688)	Stage 0 (n = 173)	Stage 1 (n = 166)	Stage 2 (n = 286)	Stages 3-4 (n = 63)	*P* Value
Age (y)	72.5 ± 11.8	67.9 ± 12.1	71.0 ± 12.1	75.2 ± 10.3	76.9 ± 12.1	0.043
Male	393 (57.1)	106 (61.3)	101 (60.8)	158 (55.2)	28 (44.4)	0.082
Body mass index (kg/m^2^)	29.0 (25.3, 33.4)	30.6 (26.6, 34.3)	29.7 (25.7, 34.7)	28.1 (25.1, 32.9)	29.2 (24.8, 33.3)	0.091
Hypertension	589 (85.6)	141 (81.5)	144 (86.7)	250 (87.4)	54 (85.7)	0.348
Hyperlipidemia	517 (75.1)	130 (75.1)	127 (76.5)	222 (77.6)	38 (60.3)	0.036
Diabetes mellitus	255 (37.1)	60 (34.7)	63 (38.0)	109 (38.1)	23 (36.5)	0.892
Coronary artery disease	355 (51.6)	75 (43.4)	87 (52.4)	156 (54.5)	37 (58.7)	0.071
Previous myocardial infarction	90 (13.1)	16 (9.2)	22 (13.3)	42 (14.7)	10 (15.9)	0.345
History of stroke	74 (10.8)	10 (5.8)	16 (9.6)	43 (15.0)	5 (7.9)	0.014
History of TIA	75 (10.9)	13 (7.5)	18 (10.8)	39 (13.6)	5 (7.9)	0.186
Chronic obstructive pulmonary disease	201 (29.2)	47 (27.2)	51 (30.7)	73 (25.5)	30 (47.6)	0.005
History of atrial fibrillation	285 (41.4)	33 (19.1)	31 (18.7)	184 (64.3)	37 (58.7)	<0.001
Creatinine (mg/dL)	1.39 ± 1.23	1.18 ± 0.60	1.27 ± 0.87	1.51 ± 1.61	1.38 ± 0.57	0.451
Chronic kidney disease						
CKD-III	173 (25.1)	36 (20.8)	39 (23.5)	79 (27.6)	19 (30.2)	0.291
CKD-IV	71 (10.3)	8 (4.6)	20 (12.0)	32 (11.2)	11 (17.5)	0.016
CKD-V/ESRD	74 (10.8)	9 (5.2)	17 (10.2)	37 (12.9)	11 (17.5)	0.019
Cirrhosis	26 (3.8)	4 (2.3)	4 (2.4)	13 (4.5)	5 (7.9)	0.149

Values are mean ± SD or n (%).

CKD = chronic kidney disease; ESRD = end-stage renal disease; TIA = transient ischemic attack.

### Echocardiographic characteristics

The mean LVEF of the cohort was 61.0% ± 8.3%. Escalating cardiac damage stage was associated with a decrease in LVEF with stages 3 to 4 patients having a mean LVEF of 57.8% ± 11.8%. With regards to AV hemodynamics, the mean peak velocity was 3.36 ± 0.29 m/s, mean peak gradient was 45.4 ± 7.9 mm Hg, average mean gradient was 25.8 ± 4.5 mm Hg, and mean AV area was 1.18 ± 0.13 cm^2^. As anticipated, patients in stages 3 to 4 had higher MV E/e’ ratios, higher LA volume indices, and higher RV systolic pressures. A summary of echocardiographic findings by cardiac damage stage is provided in Table 3.

**Table 3:** Echocardiographic Parameters

	Total Population (N = 688)	Stage 0 (n = 173)	Stage 1 (n = 166)	Stage 2 (n = 286)	Stages 3-4 (n = 63)	*P* Value^a^
LVEF (%)	61.0 ± 8.3	62.4 ± 5.7	61.2 ± 8.6	60.8 ± 8.4	57.8 ± 11.8	<0.001
Aortic valve area (cm^2^)	1.18 ± 0.13	1.18 ± 0.14	1.18 ± 0.13	1.18 ± 0.12	1.18 ± 0.14	0.460
Mean gradient (mm Hg)	25.8 ± 4.5	26.0 ± 4.6	26.2 ± 4.6	25.7 ± 4.5	24.8 ± 4.1	0.749
Peak gradient (mm Hg)	45.4 ± 7.9	45.6 ± 7.7	45.9 ± 8.0	45.2 ± 8.0	44.0 ± 7.1	0.694
Peak velocity (m/s)	3.36 ± 0.29	3.37 ± 0.28	3.37 ± 0.29	3.35 ± 0.30	3.31 ± 0.26	0.636
Dimensionless valve index	0.35 ± 0.10	0.33 ± 0.06	0.35 ± 0.09	0.38 ± 0.14	0.36 ± 0.10	<0.001
Stroke volume (mL)	90.9 ± 28.3	91.2 ± 50.0	90.6 ± 15.3	91.7 ± 15.7	86.6 ± 15.1	0.641
Stroke volume index (mL/m^2^)	46.0 ± 9.0	43.9 ± 8.7	45.5 ± 8.8	47.1 ± 9.0	47.1 ± 9.3	0.002
LV mass index (g/m^2^)	114.1 ± 36.3	99.7 ± 29.0	113.5 ± 39.7	118.9 ± 35.9	110.9 ± 32.9	0.073
Mitral valve E/e’ ratio	14.4 ± 6.1	10.2 ± 2.0	15.3 ± 5.4	15.4 ± 6.1	19.0 ± 8.9	<0.001
Left atrial volume index (mL/m^2^)	33.6 ± 13.5	24.9 ± 9.0	27.7 ± 8.1	39.6 ± 13.0	41.0 ± 16.7	<0.001
Moderate-severe mitral regurgitation	50 (7.3)	0 (0)	0 (0)	37 (12.9)	13 (20.6)	<0.001
RV systolic pressure (mm Hg)	35.0 ± 12.7	30.0 ± 8.6	31.3 ± 8.0	34.6 ± 9.7	54.8 ± 18.8	<0.001
Moderate-severe tricuspid regurgitation	49 (7.1)	0 (0)	0 (0)	0 (0)	49 (77.8)	<0.001

Values are mean ± SD or n (%).

LVEF = left ventricular ejection fraction; other abbreviations as in Table 1.

a The *P* values are calculated by ANOVA.

### Clinical outcomes

Among the entire cohort, 163 patients (23.7%) died and 105 patients (15.3%) suffered HF hospitalization across a median follow-up time of 3.6 years. The overall incidences of all-cause death and HF hospitalization were 64.4 per 1,000 person-years of follow-up and 44.3 per 1,000 person-years, respectively. The incidence of the combined primary endpoint was 93.6 per 1,000 person-years. Escalating cardiac damage stage was associated with a higher incidence of the combined primary endpoint of all-cause death and HF hospitalization, as well as each of the individual components themselves (*P* < 0.0001 for each of these endpoints).

Overall, 196 patients (28.4%) underwent AVR, including 72 patients undergoing SAVR and 124 patients undergoing TAVR. The median time to AVR was 3.1 years (IQR: 1.3-4.4), and the commonest indication for AVR was progression to severe symptomatic AS (Supplemental Table 1). The incidence of AVR overall for the cohort was 94.9 per 1,000 person-years, including incidences of 34.9 per 1,000 person-years and 60.0 per 1,000 person-years for SAVR and TAVR, respectively. Patients with stage 2 damage had a lower incidence of AVR overall, driven by a lower incidence of SAVR. Conversely, stages 0 to 1 cardiac damage were associated with an increased incidence of SAVR (*P* < 0.001). There were no statistically significant differences between groups in the incidence of TAVR, although there was a trend toward a higher incidence of TAVR (95.8 per 1,000 person-years) in patients with stages 3 to 4 damage (Table 4).

**Table 4:** Clinical Outcomes During Follow-Up by Cardiac Damage Stage

	Total Population (N = 688)	Stage 0 (n = 173)	Stage 1 (n = 166)	Stage 2 (n = 286)	Stages 3-4 (n = 63)	*P* Value^a^
All-cause death	64.4 (55.2-75.0)	38.0 (25.9-55.8)	72.0 (53.9-96.2)	61.1 (47.7-78.2)	157.6 (108.8-228.3)	<0.0001
Heart failure hospitalization	44.3 (36.6-53.6)	19.6 (11.4-33.7)	36.5 (24.0-55.5)	51.3 (38.7-67.8)	141.2 (92.5-217.7)	<0.0001
Surgical or transcatheter AVR	94.9 (82.5-109.2)	119.9 (93.5-153.8)	102.1 (78.2-133.3)	73.8 (58.0-94.0)	111.7 (66.2-188.6)	0.034
Surgical AVR	34.9 (27.7-43.9)	63.8 (45.4-89.8)	41.6 (27.4-63.2)	16.8 (10.1-27.8)	16.0 (3.99-63.8)	<0.001
Transcatheter AVR	60.0 (50.4-71.6)	56.1 (39.0-80.7)	60.5 (42.8-85.5)	57.1 (43.3-75.1)	95.8 (54.4-168.6)	0.237
Combined endpoint (all-cause death, HF hospitalization)	93.6 (82.0-106.9)	51.6 (36.9-72.2)	99.9 (77.4-128.9)	94.3 (76.6-116.0)	254.8 (184.6-351.7)	<0.0001

Values are incidence rate per 1,000 person-years of follow-up (IQR).

AVR = aortic valve replacement; HF = heart failure.

a The *P* values are calculated by chi-square test.

### Survival analyses

**Figure 1:**
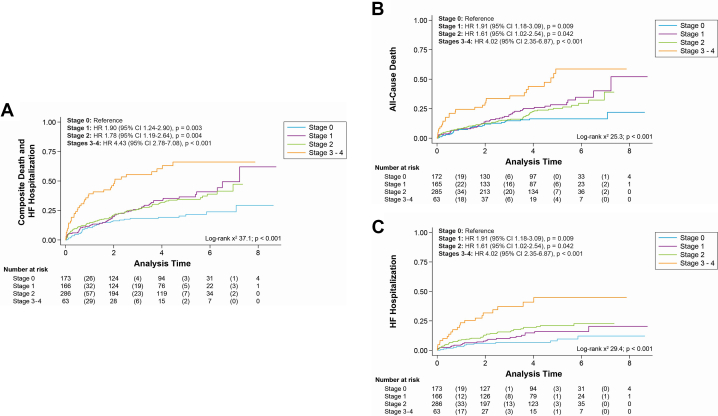
**Survival Analyses According to Cardiac Damage Stage** Higher baseline cardiac damage stage predicted a greater risk of the primary composite outcome (A), as well as the individual components of all-cause death (B) and HF hospitalization (C). HF = heart failure.

**Figure 2:**
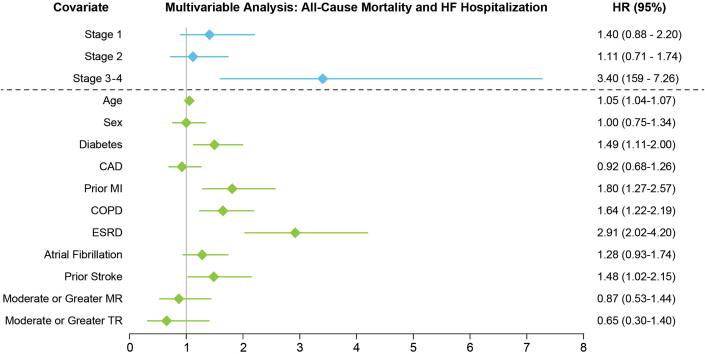
**Multivariable Analyses** There was an association between stages 3 to 4 cardiac damage and the primary composite endpoint. Age, diabetes, prior MI, COPD, ESRD, and prior stroke were also associated with the primary endpoint. CAD = coronary artery disease; COPD = chronic obstructive pulmonary disease; ESRD = end-stage renal disease; MR = mitral regurgitation; TR = tricuspid regurgitation, other abbreviations as in Figure 1.

Survival analyses for the composite primary endpoint of all-cause death and HF hospitalization, the individual components, and the secondary endpoint of AVR are included in Figure 1. Escalating cardiac damage stage was associated with a higher risk of the primary composite endpoint across all stages, with stages 3 to 4 cardiac damage conferring the highest risk (HR: 4.43: 95% CI: 2.78-7.08; *P* < 0.001) (Figure 1A). All cardiac damage stages conferred elevated risk of all-cause death, again with the greatest risk being in patients with stages 3 to 4 damage (HR: 4.02; 95% CI: 2.35-6.87; *P* < 0.001) (Figure 1B). Only stage 2 or greater cardiac damage was associated with an increased risk of HF hospitalization (Figure 1C). In multivariable analyses, only cardiac damage stages 3 to 4 were associated with an increased risk of the primary composite endpoint (HR: 3.40; 95% CI: 1.59-7.26), along with increasing age, diabetes, prior myocardial infarction, COPD, end-stage renal disease, and prior stroke (Figure 2). Cause-specific hazard of HF hospitalization or death accounting for time-updated AVR was nearly twice as high (adjusted HR: 5.93; 95% CI: 1.89-19.55; *P* = 0.003).

### Transition probabilities analyses

**Figure 3:**
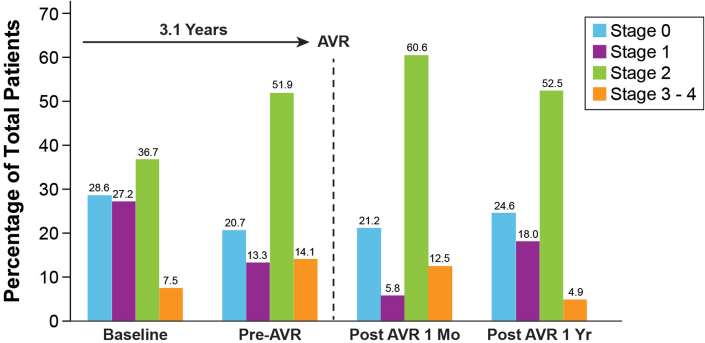
**Distributions of Patients by Cardiac Damage Stage Across Surgical Timepoints** The median time to AVR among the study cohort was 3.1 years. The percentage of patients with stage 2 and Sstages 3 to 4 cardiac damage increased while awaiting AVR. In general, patients did not tend to regress in cardiac damage following AVR. AVR = aortic valve replacement.

**Figure 4:**
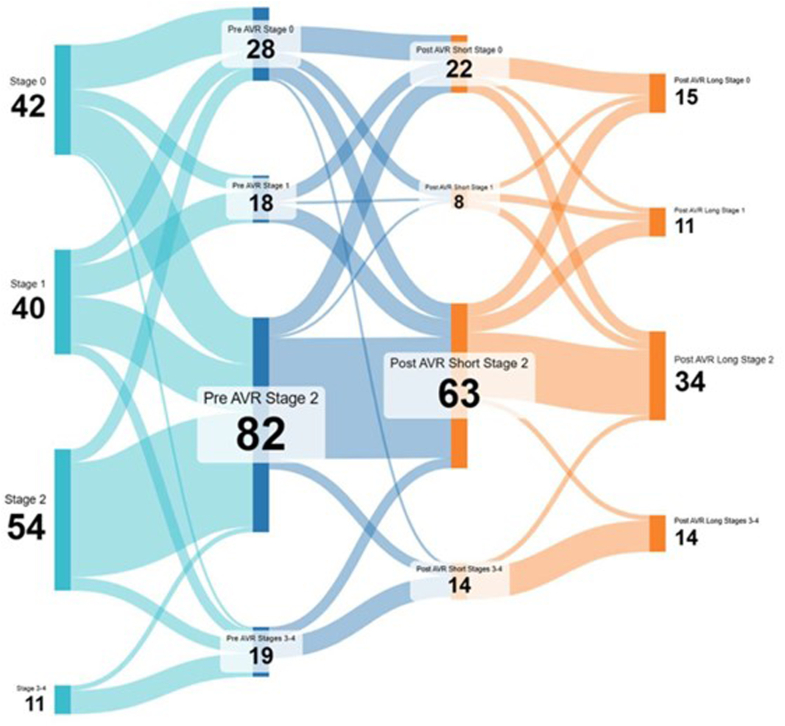
**Cardiac Damage Stage Transitions by Timepoint** Sankey diagram demonstrating the movement of patients between cardiac damage stages over time. Patients with stages 0 to 1 cardiac damage tended to escalate to stage 2 cardiac damage in the interim from baseline to time of AVR. Conversely, patients with stages 2 to 4 cardiac damage tended to remain static. After AVR, few patients regressed in cardiac damage stage. Notably, “post-AVR short” refers to 1 to 3 months follow-up, whereas “post-AVR long” refers to 1 to 3 years follow-up. Abbreviations as in Figure 3.

Distributions of patients in various cardiac damage stages at each time point are provided in Figures 3 and 4. In general, we found that in the interim period leading to AVR (median time period of 3.1 years), the proportion of patients with stages 0 to 1 damage decreased, whereas the proportion of patients in stages 2 and 3 to 4 damage increased, and the number of patients with stage 2 damage remained elevated even out to 1 to 3 years post-AVR (Figure 3). During the waiting periods from moderate AS diagnosis to AVR, a patient in stage 0 had a 60% probability of progression to stages 1 to 3, and a patient in stage 1 had 55% probability to progress to stages 2 to 3.

In contrast, patients in stages 3 to 4 had more than 70% probability to remain in their respective stages. Very few patients reverse remodeled to a lower cardiac damage stage following AVR, although there was a 22 to 33% probability for stages 3 to 4 patients to improve to stage 2. Patients in pre-AVR stage 2 had a 20% probability of reverse remodeling to stages 0 to 1 (Figure 4). Interestingly, the presence or absence of LA dilatation was the most common driver of both worsening and improvement of cardiac damage at each of the 3 AVR timepoints (pre-AVR, 1-3 month post-AVR, and 1-3 year post-AVR) (Supplemental Tables 2A to 2).

## Discussion

Using a relatively novel form of cardiac structural and functional damage assessment via a formal staging classification validated in severe AS patients,the present findings demonstrate in the moderate AS population, there remains a stepwise increased risk of all-cause mortality and HF hospitalization. In addition, the time period from moderate AS diagnosis to ultimate AVR yielded progressive accumulation of greater degrees of cardiac damage. Although AVR was beneficial in reducing the incidence of later-stage cardiac damage in the postprocedural period, the net result of treatment delay and eventual AVR culminated in a greater proportion of patients harboring more cardiac damage compared with their baseline status at the time of moderate AS diagnosis. As we await pivotal trials assessing TAVR in symptomatic moderate AS, the present analysis supports the notion of moderate AS patients potentially benefiting from earlier AVR at the expense of worsening cardiac damage (Central Illustration).

**Central Illustration:**
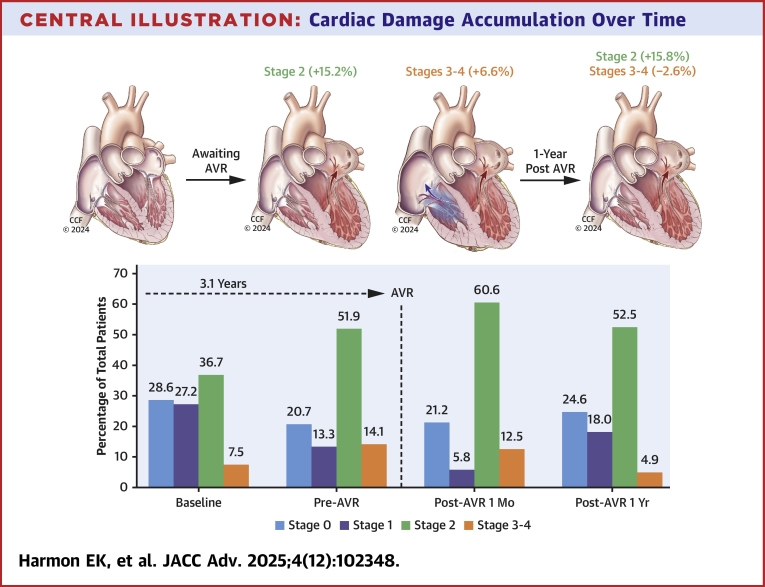
**Cardiac Damage Accumulation Over Time** Cardiac damage tends to accumulate in patients with moderate AS while awaiting AVR, with escalating numbers of patients found to have stages 2 and 3 to 4 damage. Damage partially-regresses following AVR but stage 2 damage tends to persist. AVR = aortic valve replacement.

An increasing body of evidence suggests that patients with moderate AS are more vulnerable for cardiac events than has been historically appreciated. A recent meta-analysis of over 400,000 patients (including over 11,000 with moderate AS) found that moderate AS conferred a 2.5-fold increased risk of all-cause death even after adjusting for age, sex, and LVEF.^15^ A second meta-analysis of over 12,000 patients with moderate AS described an associated mortality rate of 9% per year.^16^ In addition, in a real-world database including over 14,000 patients with moderate AS, the associated 4-year mortality of patients who did not undergo AVR was reported to be 33.5%.^7^ Thus, moderate AS does not appear to be a benign entity, which our results further confirm with an overall cohort mortality of 23.7% over a median follow-up period of 3.6 years.

The present data also suggest that current definitions of “moderate” and “severe” AS are imperfect, and perhaps additional methods of risk stratification beyond severity grading (that are currently inherently based on binary cut points) are warranted. In reality, AS exists on a spectrum which is defined not purely by valvular hemodynamics alone, but instead the complex interplay between the stenotic valve itself and multiple secondary factors including the contractility and compliance of the left ventricle, remodeling of the left atrium and pulmonary vasculature, aortic stiffness, and arterial hypertension.^1^^,^^17^ As a result, there has been an increased interest in identifying methods by which the risk assessment of patients with AS may be further refined. The present analysis provides additional validation of prognosticating hard clinical events according to cardiac damage stage, yet in the moderate AS population.

It is important to clarify that the relationship between moderate AS and cardiac damage may not be causal. Although certain pathologic changes such as LV hypertrophy and LA dilatation may be attributable to moderate AS per se, other damage markers may reflect other concomitant comorbidities. For example, the present analysis demonstrates that escalating cardiac damage stage did associate with multiple medical comorbidities including COPD and advanced kidney disease. As such, escalating cardiac damage should be interpreted as identifying the vulnerable substrate unlikely to tolerate moderate AS over time, and the key question is whether normalizing AV function portends clinical benefit in such comorbid individuals.

Data from the recently-published TAVR UNLOAD trial suggest that TAVR for moderate AS in such vulnerable patients may not be warranted.^18^ Among patients with chronic systolic HF (LVEF 20% to 50%), NYHA functional class II-IV symptoms, and moderate AS, TAVR failed to significantly improve the primary hierarchical composite endpoint of all-cause death, disabling stroke, disease-related hospitalization and HF hospitalization equivalents, and change from baseline in the Kansas City Cardiomyopathy Questionnaire Overall Summary Score compared to patients managed with optimal medical therapy for HF alone. However, this trial had several important limitations which should be considered. With only 178 patients included, the study itself was underpowered to detect significant differences in the primary endpoint. Perhaps equally important, a high proportion (43%) of patients in the medical therapy and surveillance arm ultimately required TAVR at 12 months. This would support the notion that certain vulnerable phenotypes may accelerate in their disease progression at faster rates than would otherwise be expected. In fact, the authors themselves note that a cardiac damage framework may be useful in identifying patients most vulnerable to the adverse afterload imposed by moderate AS.

Our analysis suggests that delaying AVR correlates with accumulating greater levels of cardiac damage (as evidenced by a high [55% to 60%] probability of progression from stages 0 to 1 to a higher stage over a median of 3 years), and that AVR did seem to favorably reverse greater degrees of accumulated cardiac damage (as evidenced by 22% to 33% probability of reverse remodeling from stages 3-4 post-AVR compared with pre-AVR). It remains notionally feasible that the comorbidities contributing to cardiac damage are less “reversible” than moderate AS per se, and that such comorbidities may not only condition the heart to be more sensitive to progressive cardiac damage, but also susceptible to deriving benefit from AVR in the presence of moderate AS.

From a mechanistic perspective, although regression in LV hypertrophy and improvement in LV systolic function tend to occur following AVR, myocardial fibrosis itself (and subsequent diastolic dysfunction) may persist.^19^^,^^20^ In transition analyses, we found that patients with either no cardiac damage (stage 0) or LV damage (stage 1) tended to increase in damage stage in the time preceding their AVR, and even after AVR, very few patients re-entered a lower damage stage. This may very well reflect fibrotic changes occurring within the myocardial extracellular matrix in these patients as their AV disease progresses, changes which are not necessarily reversible. This may provide further evidence for an earlier, more aggressive approach to treating moderate AS irrespective of underlying cardiac damage stage, with those at an earlier cardiac damage stage standing to derive the most benefit.

### Study limitations

Our study has several important limitations. Given its retrospective, single-center design, the present analysis is prone to unavoidable selection bias. This may be reflected in part by the high proportion of AF in this cohort overall (41.4%), as well as the unexpected finding that stroke volume remained preserved across all cardiac damage stages. This was even true of patients with stages 3 to 4 damage, despite high prevalences of AF (58.7%), MR (20.6%), and TR (77.8%), all of which would be expected to reduce stroke volume. One explanation for this finding might be the preservation of biventricular function in the vast majority of patients. Another explanation might be the strict moderate AS criteria applied in this study which would not have included paradoxical low-flow, low-gradient AS. Regardless, the potential for selection bias limits the generalizability of our results.

In addition, our transitional probability analyses were limited to only descriptive findings. This is owing to the fact that cardiac damage stages applied at various timepoints were dependent on the timing of AVR (eg 1-3 months pre-AVR, 1-3 months post-AVR, and 1-3 years post-AVR). Therefore, we could not adjust for this variability in a way that would allow for assignment of cardiac damage stages at similar time points for patients who did not undergo AVR (ie, there is not a comparator group). Finally, as a quaternary referral center, some patients were lost to follow-up despite efforts to review outside labs, echocardiography reports, and clinical encounters. A strength of our study, however, was the strict application of the moderate AS criteria to provide a more enriched population. Definitions of moderate AS in the literature vary dramatically, and this may in part explain the heterogeneity of outcomes observed across studies.^15^ Not only were our criteria consistent with current guidelines,^1^ but also reflect inclusion criteria similar to those of 2 major ongoing TAVR trials for moderate AS (EXPAND TAVR II [NCT05149755] and PROGRESS [NCT04889872]).

## Conclusions

The present data demonstrate that increasing cardiac damage stage at the time of moderate AS diagnosis is associated with an incrementally higher risk of all-cause mortality and HF hospitalization. In addition, patients with moderate AS appear to progress in cardiac damage staging leading up to AVR, an effect that does not seem to completely diminish postoperatively. Further studies are needed to identify particularly vulnerable patients with moderate AS who may benefit from earlier therapies including AVR.

Perspectives **COMPETENCY IN PATIENT CARE AND PROCEDURAL SKILLS:** This study provides a relatively novel risk assessment tool for patients with moderate AS. Consideration of cardiac damage stage may aid in the early identification of particularly vulnerable moderate AS patients.

**TRANSLATIONAL OUTLOOK:** There is an increasing body of evidence that the current standards of risk stratification in AS are insufficient in capturing the complex interplay between valvular hemodynamics, cardiac structure and function, and medical comorbidities of individual patients. As such, there may be vulnerable patients with even “moderate” AS who may benefit from more aggressive medical and procedural therapies. Future studies should include standardized definitions of moderate AS and attempt to further refine our understanding of which patients may stand to benefit the most from earlier intervention.

## Funding support and author disclosures

The authors have reported that they have no relationships relevant to the contents of this paper to disclose.
