# Supplementary material for: Cardiac Damage Staging, Moderate Aortic Stenosis, and the Impact of Aortic Valve Replacement
Source: JACC Adv. 2025 Nov 15;4(12):102348. doi: 10.1016/j.jacadv.2025.102348 (PMC12664025; doi:10.1016/j.jacadv.2025.102348)
Supplement: Supplemental material [file mmc1.docx]

| **SUPPLEMENTARY TABLE 1 Transcatheter and Surgical AVR Procedural Details** | | | | |
| --- | --- | --- | --- | --- |
|  |  | **TAVR**  **(n = 124)** | **SAVR**  **(n = 72)** | **p Value*** |
| **Baseline Cardiac Damage Stage**  0  1  2  3-4 | | 29 (23.4)  32 (25.8)  51 (41.1)  12 (9.7) | 33 (45.8)  22 (30.6)  15 (20.8)  2 (2.8) | 0.001 |
| **Indication for AVR/OHS**  Severe symptomatic AS  Severe symptomatic AI  Multivessel CAD  Aortic aneurysm | | 124 (100)  --  --  -- | 58 (80.6)  3 (4.2)  7 (9.7)  4 (5.6) | <0.0001 |
| **Valve type**  Balloon-expandable  Self-expanding  Mechanically-expanded  Bioprosthetic  Stented  Stentless  Sutureless  Mechanical  Aortic valved conduit | | 108 (87.1)  15 (12.1)  1 (0.8)  --  --  --  --  -- | --  --  --  65 (90.3)  0 (0.0)  1 (1.4)  5 (6.9)  1 (1.4) | <0.0001 |
| **Valve size**  19 mm  20 mm  21 mm  22 mm  23 mm  25 mm  26 mm  27 mm  29 mm  34 mm | | --  1 (0.8)  --  --  30 (24.2)  0 (0.0)  60 (48.4)  1 (0.8)  26 (21.0)  7 (5.6) | 1 (1.4)  --  12 (16.7)  1 (1.4)  20 (27.8)  23 (31.9)  --  12 (16.7)  2 (2.8)  -- | <0.0001 |
| **Concomitant Surgical Procedure**  Mitral valve repair/replacement  Tricuspid valve repair/replacement  Coronary artery bypass grafting  Aortic repair/replacement  Surgical Maze  LAA clipping/excision  Myectomy | | --  --  --  --  --  --  -- | 7 (9.7)  4 (5.6)  29 (40.3)  20 (27.8)  8 (11.1)  13 (18.1)  2 (2.8) | --  --  --  --  -- --  -- |

| **Supplementary Table 2A. Frequency of Criteria Accounting for Stage Change at Timepoint Pre-AVR** | | | | | | | | | |
| --- | --- | --- | --- | --- | --- | --- | --- | --- | --- |
|  | **LVMI** | **E/e’** | **LVEF** | **LAVI** | **MR** | **AF** | **RVSP** | **TR** | **RV** |
| Increased Stage | 2 (4.2%) | 4 (8.5%) | 0 (0%) | 32 (68.0%) | 1 (2.1%) | 0 (0%) | 4 (8.5%) | 3 (6.4%) | 1 (2.1%) |
| Decreased Stage | 1 (11.1%) | 2 (22.2%) | 0 (0%) | 4 (44.4%) | 0 (0%) | 0 (0%) | 1 (11.1%) | 1 (11.1%) | 0 (0%) |

| **Supplementary Table 2B. Frequency of Criteria Accounting for Stage Change at Timepoint 1-Month Post-AVR** | | | | | | | | | |
| --- | --- | --- | --- | --- | --- | --- | --- | --- | --- |
|  | **LVMI** | **E/e’** | **LVEF** | **LAVI** | **MR** | **AF** | **RVSP** | **TR** | **RV** |
| Increased Stage | 1 (2.5%) | 4 (10.0%) | 0 (0%) | 17 (42.5%) | 2 (5.0%) | 6 (15.0%) | 1 (2.5%) | 9 (22.5%) | 0 (0%) |
| Decreased Stage | 4 (21.1%) | 1 (5.3%) | 0 (0%) | 6 (31.6%) | 1 (5.3%) | 0 (0%) | 4 (21.1%) | 2 (10.5%) | 1 (5.3%) |

| **Supplementary Table 2C. Frequency of Criteria Accounting for Stage Change at Timepoint 1-Year Post-AVR** | | | | | | | | | |
| --- | --- | --- | --- | --- | --- | --- | --- | --- | --- |
|  | **LVMI** | **E/e’** | **LVEF** | **LAVI** | **MR** | **AF** | **RVSP** | **TR** | **RV** |
| Increased Stage | 0 (0%) | 1 (5.3%) | 0 (0%) | 9 (47.4%) | 0 (0%) | 2 (10.5%) | 1 (5.3%) | 4 (21.1%) | 2 (10.5%) |
| Decreased Stage | 1 (6.7%) | 2 (13.3%) | 1 (6.7%) | 8 (53.3%) | 0 (0%) | 0 (0%) | 1 (6.7%) | 1 (6.7%) | 1 (6.7%) |

Cause-specific Cox censored at AVR

. stcox i.stage3 age i.sex i.DM i.CAD i.MI i.COPD i.CKD5_ESRD i.AF i.stroke i.MR2 i.TR2

Failure _d: compHFHdead==1

Analysis time _t: tAVRdeathHFH

ID variable: study_id

Iteration 0: Log likelihood = -457.64584

Iteration 1: Log likelihood = -420.41566

Iteration 2: Log likelihood = -412.28882

Iteration 3: Log likelihood = -412.17874

Iteration 4: Log likelihood = -412.17869

Refining estimates:

Iteration 0: Log likelihood = -412.17869

Cox regression with Breslow method for ties

No. of subjects = 534 Number of obs = 534

No. of failures = 81

Time at risk = 773,126

LR chi2(14) = 90.93

Log likelihood = -412.17869 Prob > chi2 = 0.0000

------------------------------------------------------------------------------

_t | Haz. ratio Std. err. z P>|z| [95% conf. interval]

-------------+----------------------------------------------------------------

stage3 |

1 | 2.626177 1.011858 2.51 0.012 1.234124 5.588425

2 | 1.59248 .6353199 1.17 0.243 .7285967 3.480654

3 | 5.932033 3.609108 2.93 0.003 1.800214 19.54713

|

age | 1.033496 .0129648 2.63 0.009 1.008395 1.059221

|

sex |

Male | .936186 .2269035 -0.27 0.786 .5821788 1.505456

1.DM | 1.474188 .3624026 1.58 0.114 .9105427 2.386742

1.CAD | 1.487059 .4075987 1.45 0.148 .8689927 2.544721

1.MI | 2.49661 .6881888 3.32 0.001 1.454513 4.285326

1.COPD | 1.757325 .4270737 2.32 0.020 1.091412 2.829536

1.CKD5_ESRD | 3.008255 .9390143 3.53 0.000 1.631607 5.546432

1.AF | 1.680227 .4400105 1.98 0.048 1.005679 2.807223

1.stroke | 2.012785 .6336851 2.22 0.026 1.085959 3.730621

1.MR2 | .974094 .4244332 -0.06 0.952 .4146813 2.288165

1.TR2 | .5813734 .3605118 -0.87 0.382 .1724336 1.960146

------------------------------------------------------------------------------

. stcox i.stage age i.sex i.DM i.CAD i.MI i.COPD i.CKD5_ESRD i.AF i.stroke i.MR2 i.TR2

Failure _d: compHFHdead==1

Analysis time _t: tAVRdeathHFH

ID variable: study_id

Iteration 0: Log likelihood = -457.64584

Iteration 1: Log likelihood = -433.71775

Iteration 2: Log likelihood = -412.59576

Iteration 3: Log likelihood = -411.74257

Iteration 4: Log likelihood = -411.74089

Iteration 5: Log likelihood = -411.74089

Refining estimates:

Iteration 0: Log likelihood = -411.74089

Cox regression with Breslow method for ties

No. of subjects = 534 Number of obs = 534

No. of failures = 81

Time at risk = 773,126

LR chi2(15) = 91.81

Log likelihood = -411.74089 Prob > chi2 = 0.0000

------------------------------------------------------------------------------

_t | Haz. ratio Std. err. z P>|z| [95% conf. interval]

-------------+----------------------------------------------------------------

stage |

1 | 2.639167 1.016186 2.52 0.012 1.240852 5.613241

2 | 1.622349 .6473979 1.21 0.225 .7421171 3.546631

3 | 4.115196 3.206065 1.82 0.069 .8937923 18.94717

4 | 9.887762 7.712872 2.94 0.003 2.143507 45.61116

|

age | 1.033219 .012957 2.61 0.009 1.008134 1.058929

|

sex |

Male | .9160357 .2234313 -0.36 0.719 .5679301 1.477508

1.DM | 1.44422 .3571201 1.49 0.137 .8895123 2.344847

1.CAD | 1.527282 .4202393 1.54 0.124 .8906499 2.618977

1.MI | 2.384349 .6729591 3.08 0.002 1.371281 4.145845

1.COPD | 1.741008 .4235064 2.28 0.023 1.080794 2.80452

1.CKD5_ESRD | 3.007135 .9446549 3.50 0.000 1.624644 5.566056

1.AF | 1.621283 .4304874 1.82 0.069 .9634865 2.728175

1.stroke | 2.012017 .6333784 2.22 0.026 1.085614 3.728962

1.MR2 | .9232516 .4136919 -0.18 0.859 .3836299 2.221916

1.TR2 | .839638 .6585423 -0.22 0.824 .1805013 3.905744

------------------------------------------------------------------------------
